# Supplementary figures and images for: Tick species diversity and potential distribution alternation of dominant ticks under different climate scenarios in Xinjiang, China
Source: PLoS Negl Trop Dis. 2024 Apr 29;18(4):e0012108. doi: 10.1371/journal.pntd.0012108 (PMC11081513; doi:10.1371/journal.pntd.0012108)

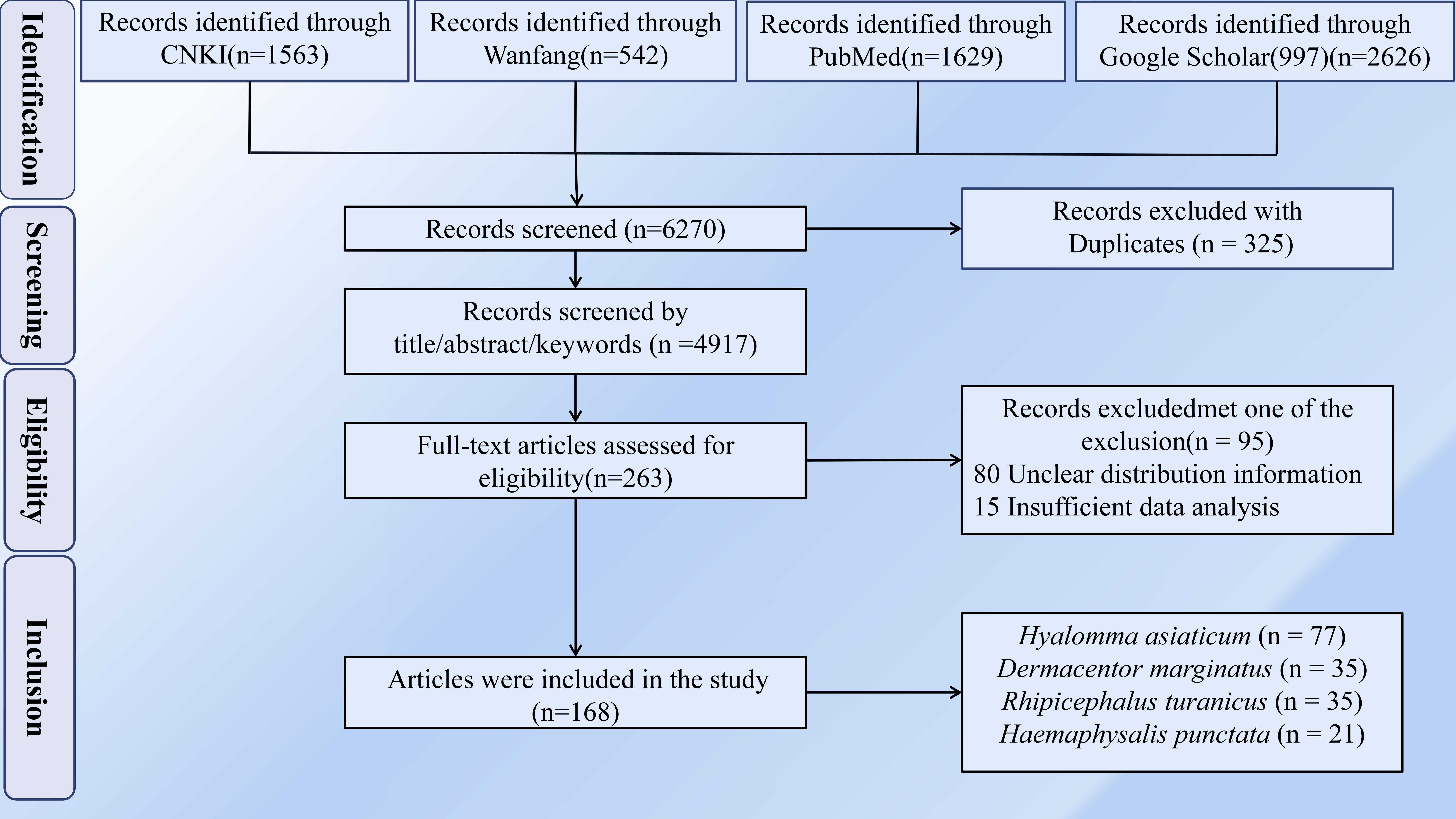

Supplement: S1 Fig — (TIF) [file pntd.0012108.s004.tif]
