# Supplementary material for: Tick species diversity and potential distribution alternation of dominant ticks under different climate scenarios in Xinjiang, China
Source: PLoS Negl Trop Dis. 2024 Apr 29;18(4):e0012108. doi: 10.1371/journal.pntd.0012108 (PMC11081513; doi:10.1371/journal.pntd.0012108)
Supplement: Attachment — Submitted filename: Responses+to+the+review+comments0314(1).docx [file pntd.0012108.s005.docx]

Dear Editorial Assistant of PLOS Neglected Tropical Diseases:

On behalf of my co-authors, we thank you very much for allowing us to revise our manuscript; we appreciate the editor and reviewers very much for their positive and constructive comments and suggestions on our manuscript "Tick species diversity and potential distribution alternation of dominant ticks under different climate scenarios in Xinjiang, China".

We have studied the reviewer’s comments carefully and made revisions marked in red in the revised MS. We have tried our best to revise our manuscript according to the comments. Attached is the revised version, which we would like to submit for your kind consideration.

We would like to thank you and the reviewers for your comments on our paper. I am looking forward to hearing from you.

Thank you and best regards.

Yours sincerely,

Xinyu Feng

E-mail: [fengxinyu2013@163.com](mailto:fengxinyu2013@163.com)

Reviewer's Responses to Questions

Reviewer #1: Readers likely do not know what the “China National Knowledge Infrastructure” provides – so this needs to be explained.

**Response: We thank the reviewer for the kind comments. we have modified it to “CNKI (China National Knowledge Infrastructure) databases (the largest and most comprehensive academic database for Chinese scholarly publications)” in line 86-87.**

In some cases “the coordinates of the distribution points were determined using the coordinate picking function in Google Maps based on the geographical location” but this needs more explanation – there must be some criterion on which to base use of such articles – maybe if they mentioned a particular town or municipality?

**Response: We extract coordinate data directly if the article contained the values. We extract coordinate data based on the geographical locations using the coordinate picking function in Google Maps.**

“Only one distribution point was retained in each 10 km × 10 km grid” – what does this mean as in the previous sentence it was mentioned that a buffer of 10km was used?

**Response: Sorry for the confusion, we have modified it to “Only one distribution point was retained in each buffer zone”.**

A correlation coefficient of 0.9 is a very low bar for eliminating possibly correlated explanatory variables. Often 0.6 is used.

**Response: Thank you again for your valuable suggestions. We have referred to the literature that used maxent to predict tick distribution, and most of them adopted 0.9 to eliminate possibly correlated explanatory variables. A correlation coefficient of 0.9 suggests a very strong positive linear relationship and would be considered high in most contexts. At the same time, we realize that 0.9 can also lead to overfitting and reduced model generalizability.**

**References:**

**[1] Sánchez Pérez M, Feria Arroyo T P, Venegas Barrera C S, et al. Predicting the Impact of Climate Change on the Distribution of Rhipicephalus sanguineus in the Americas[J]. Sustainability, 2023, 15(5): 4557.**

**[2] Yao, Xiaoyan., Tian, Na., Ma, Ben. et al. Climate change on China's small fan head the influence area of tick [J]. Chinese journal of schistosomiasis control, 2021 (03) : 267-273.**

**[3] Ma, Ben., Ma, Xiaoyu., Zhang, Yi., et al. Prediction of suitable habitats of Ixodes persulcatus in China [J]. Chinese journal of schistosomiasis control, 2021 (02) : 169-176.**

The WorldClim database is used for climate data for the MaxEnt model and spatial resolution of this is provided. However WorldClim is an interpolation of climate station data – so the article needs to identify how many climate stations in the study region, that contributed to WorldClim are present in the study region. Confidence in the climate data used would be very different if there were 3 or 300 meteorological stations providing data.

**Response:** According to the official introduction of the Xinjiang Meteorological Bureau, Xinjiang has 105 manned weather stations with complete business and a high level of modernization, and 505 unattended automatic stations. It has built 8 lightning locators, 1 GPS/MET water vapor flux station, 12 solar radiation observation stations, 7 acid rain observation stations, 7 dust storms, atmospheric composition observation stations, and 17 wind towers. The MODIS Polar Orbit Satellite receiving system is built on the basis of China Fengyun Meteorological Satellite and NOAA satellite receiving system.

A table identifying the geographic resolution of explanatory variable data and tick data would help the reader understand the relationships amongst these data.

**Response: We thank the reviewer for the kind comments. We provided Climate and Geographic Variables and tick data in Additional Table 1 which hope to help the reader understand the relationships amongst these data.** **If reader need detailed record data, they can further contact us through email and other forms to obtain, we are more than happy to provide.**

Reviewer #2: Please provide the languages and search terms used for the literature search described in lines 84-87.

**Response：We have modified it to “used the keywords "Xinjiang" and "tick" to search”.**

Line 114, please change "the main factor" to "an important factor". Climate is not the single main factor influencing tick distribution.

**Response：We are very grateful for your comments. We have changed the relevant content in the revised manuscript, as you kindly suggested.**

Reviewer #1: It is stated that there was a ‘top 4’ variables used – but only 3 are mentioned.

It seems that the only climatic determinant of tick distributions is precipitation, which is much more uncertain in climate model outputs than temperature.

**Response: Sorry for the confusion, we have modified it to “the top 4 environmental variables are the Precipitation of Wettest Month (Bio13), Precipitation Seasonality (Bio15), Population Density (PD) and Isothermality (Bio3), which explained a significant quotient of all variables. The four species of ticks are mainly influenced by Precipitation of Wettest Month (Bio13) and Population Density (PD), and the contribution rates are all far more than 15%. ”**

Reviewer #2: Results are appropriate

**Response: Thank you for your positive comments.**

Reviewer #1: Throughout it needs to be clear that collecting information from literature only provides possible distributions (the article does not provide an ‘up-to-date map of tick distribution in Xinjiang’ as mentioned in the abstract) – not actual distributions, and that extensive field study is needed to confirm or deny findings in the article.

**Response: Thank you again for your valuable suggestions. We have modified it to “preliminary map of tick distribution in Xinjiang”. Our analysis identifies areas where ticks are likely to be found, based on ecological, climatic, and geographical factors reported in the literature. However, due to the dynamic nature of tick populations and potential changes in their habitats over time, the actual distributions may differ from our findings. We strongly emphasize the need for comprehensive field studies to verify and update the distributions of tick populations in Xinjiang.**

Reviewer #2: Discussion and conclusion are appropriate

**Response: Thank you for your positive comments.**

Reviewer #1: (No Response)

Reviewer #2: The figures are a little blurry and authors will need to upload high-resolution map images.

**Response: Thank you again for your valuable suggestions. We have provided high-resolution map images.**

Overall, the English writing and grammar are good; however, small editorial changes will need to be made. For example, several terms are capitalized when they should be lowercase.

**Response: Sorry for the confusion, we scrutinized and revised some of the terms in the manuscript.**

--------------------

Reviewer #1: Major comments:

The article obtains from the literature possible tick distributions, and from these, possible future distributions are projected. Throughout it needs to be clear that collecting information from literature only provides possible distributions (the article does not provide an ‘up-to-date map of tick distribution in Xinjiang’ as mentioned in the abstract) – not actual distributions, and that extensive field study is needed to confirm or deny findings in the article.

**Response: Sorry for the confusion, we have modified it to “preliminary map of tick distribution in Xinjiang”. Our analysis identifies areas where ticks are likely to be found, based on ecological, climatic, and geographical factors reported in the literature. However, due to the dynamic nature of tick populations and potential changes in their habitats over time, the actual distributions may differ from our findings. We strongly emphasize the need for comprehensive field studies to verify and update the distributions of tick populations in Xinjiang.**

The treatment of projected future climate is very limited. There is only one greenhouse gas concentrations scenario (when normally a range would be included), and no accounting for variations amongst models in the CMIP6 ensemble. These sources of variations should be included.

**Response: We appreciate your feedback regarding our study's approach to modeling future climates. Although we initially chose this approach due to certain research constraints and focus areas, we recognize the significance of a more comprehensive treatment that encompasses the full range of potential future climates. Thank you for pointing out the critical limitations in our approach to projecting future climate conditions. We used SSP245 scenario under the BCC-CSM2-MR Model of the Sixth Coupled model Intercomparison Project (CMIP6).** **SSP245 represents an upgrade of the RCP4.5 scenario based on SSP2, a medium forcing scenario. From the perspective of future greenhouse gas emissions and concentration trends, the RCP 4.5 scenario peaked in 2040 and stabilized by 2080（CO2 emissions in CMIP6 scenarios : In 2020, it will be 40.65, in 2040, 44.25, in 2060, 40.20, in 2080, 26.84, and in 2100, 9.68）.**

How results from MaxEnt models are used to provide climate envelopes for tick species, that are then used to project future tick distributions, is poorly explained in the methods.

MaxEnt is only one amongst a number of methods that are used to estimate determinants of the ecological niche of species – explanation of the selection of this method, and rejection of others, is needed.

**Response: Thank you very much for your circumspection. We opted for MaxEnt after evaluating several alternative methods, including Bioclim, GARP (Genetic Algorithm for Rule-set Prediction), and SDM (Species Distribution Modelling). These methods also offer valuable insights into species' ecological niches; however, our preliminary analyses suggested that MaxEnt provided the most accurate and robust predictions for our specific data set and study goals. We have added and explained in the methods that “The Maximum Entropy (MaxEnt) model is a widely used methodology in ecological niche modeling that relies on the fundamental theory of species ecological niches. The model utilizes information on the spatial distribution of species occurrence data and relevant environmental background data to estimate the probability distribution with the maximal entropy value, thereby providing an estimation of the potential distribution of a given species.” in line 133-136. However, it is important to note that the model only provides potential areas where a given species may survive, which may not necessarily represent actual distributions or species abundance as discussed in the present study. This is also a limitation of the current Maxent model.**

Minor comments

The article needs a check on English language – overall it is very good but the abstract needs particular attention.

Abstract:

Ticks are not reservoirs.

Data, being plural, ‘were’ rather than ‘was’.

Some specifics are needed on key tick species rather than simply saying they ‘fluctuated’.

**Response: Thank you for your positive comments and valuable suggestions. We have changed according to your suggestion. We have modified it to “ a vector of pathogens”.**

**We have modified it to “The distribution datas were extracted, corrected, and deduplicated”.**

**We have modified it to “In the four future periods, the range of medium and high suitable areas of the potential distribution area of the four ticks will expand to the northwest, and new suitability areas being distributed in Altay, Changji Hui Autonomous Region, and other local areas”.**

Introduction:

Please include the tick species involved in TBEV-2871 transmission.

It is true that “Previous studies show that the distribution of ticks is closely related to the natural environment and exhibits distinct regional and seasonal characteristics” but the literature cited is very limited.

The objective “to depict the distribution map of tick species in Xinjiang over a period of 60 years” is not correct – the objective is to identify possible distributions from 60 years of literature.

**Response: (1) Thank you again for your valuable suggestions. We have added the tick species involved in TBEV-2871 transmission (*Ixodes pavlovskyi*).**

**(2) We have added references to this content [12-17].**

**(3) we have changed according to your suggestion. We have modified it to “identify possible distributions of tick species in Xinjiang over a period of 60 years”.**

Reviewer #2: The authors describe a new multi-country highway system that has the potential to inadvertently introduce new tick species into the region of Xinjiang, China. This article is a new contribution to the scientific literature and has broader implications for other countries who are planning large-scale transportation infrastructure projects. The authors leveraged the historical literature to inform climate change models to predict future expansion of vector-specific habitable areas in the region.

**Response: Thank you for your positive comments**
